# Blast resistance of RC tubular structure under internal ANFO explosion

**DOI:** 10.1038/s41598-022-26062-9

**Published:** 2022-12-16

**Authors:** Seung-Jai Choi, Tae-Hee Lee, Norhazilan Md. Noor, Jang-Ho Jay Kim

**Affiliations:** 1 School of Civil and Environmental Engineering, Yonsei University, 50 Yonsei-Ro, Seodaemun-Gu, Seoul, 03722 South Korea; 2 School of Civil and Environmental Engineering, Yonsei University, 50 Yonsei-Ro, Seodaemun-Gu, Seoul, 03722 South Korea; 3 Department of Structure and Material, Universiti Teknologi Malaysia, 81310 Skudai, Johor Malaysia; 4 School of Civil and Environmental Engineering, Yonsei University, 50 Yonsei-Ro, Seodaemun-Gu, Seoul, 03722 South Korea

**Keywords:** Civil engineering, Mechanical engineering

## Abstract

The degree of structural damage is significantly more severe when a blast occurs inside than outside of a structure. However, existing designs for RC structures such as reinforced concrete containment vessels (RCCV) do not include design features to protect the structure for internal blast. Therefore, the internal blast resistance capacity of RC structures is evaluated by performing internal blast tests on RC tubular structures. The main objective of the study was to observe and document the basic structural behavior data obtained from internal detonation tests. ANFO explosive charge weights of 15.88, 20.41, 22.68 and 24.95 kg were selected for a charge detonating at a cross section center of the mid-span of the specimen, giving a standoff distance to the inner wall surface of 1000 mm. The data acquisitions include blast pressure, deflection, strain, and crack pattern. When the explosive charge weight increased from 15.88 to 24.95 kg, the peak incident pressure and time duration increased from 0.1718 to 0.3394 MPa and from 5.856 to 5.981 ms, respectively. Then, the test data were used to predict the internal charge weight required to fail a real scale RCCV using simple assumptions and the test data. The results of the study are discussed in detail in the paper.

## Introduction

Reinforced concrete (RC) is a most widely used structure type for construction of buildings and infrastructure. However, the drawback of RC structure is its brittle failure characteristic due to macro tensile cracking and spalling damage. In particular, when an instantaneous blast load is applied to a RC member, it fails catastrophically with a sudden loss of load-bearing capacity. When an extreme load such as blast and impact is applied to a RC structure, its resistance depends greatly on its stiffness and load bearing capacity. If RC structure experiences cracks and inelastic deflections from a blast load, the structure is no longer serviceable for its intended design usage. To analyze the structural stiffness and load-bearing capacity of a structural member under extreme blast loading, test and simulations need to be performed. Also, because of worldwide concern for terrorism and accidents, the accurate prediction of structural resistance to blast loading is urgently needed presently. Despite difficulties in conducting field blast tests, various researchers have performed blast load tests and simulations on scale models of actual RC structures. Various researchers have established structural models and constitutive equations according to blast loading^6,10,13,14,16^. However, among various types of blast loading, a blast occurring inside of a structure is a most serious blast type due to its continuous reflection of blast pressures inside of an enclosed space. Also, during an internal blast test, internal blast pressure measurement is nearly impossible since pressure gauges installed inside the enclosed space are damaged by the reflected blast pressures. For this reason, there is very limited number of test data available on internal blast characteristic and its structural effect.

South Korea is the sixth-largest nuclear energy-producing nation in the world, having 24 servicing nuclear power plants (NPPs) with its first NPP built in 1978. Approximately, 40% of all electricity generated in Korea is produced from NPPs. Containment vessels in Kori 1 and 2 NPP are RC containment vessel (RCCV) type. Since, Kori 1 and 2 NPP are in the process of being decommissioned, there is a realistic possibility of internal blast accident occurring during demolition work, which must be pre-analyzed prior to the work. Despite the danger of extreme accident scenario occurring in the RCCVs, the current design codes do not have detailed extreme disaster design guidelines on blast and collision loads on NPP structure^3^. However, several previous NPP disasters such as Chernobyl and Fukushima NPP revealed the vulnerability of NPP structure from extreme disaster scenarios. Therefore, in this study, a scaled down model of a RCCV was designed and fabricated for internal blast test. Then, the test data are obtained to be used for RCCV model calibration for internal blast structural simulation. Finally, the calibrated model is used for real scale RCCV analysis to predict and calculate the internal blast charge weight needed to fail a RCCV.

## Literature review

Literature review of researches on extreme disaster prevention for RC structures, showed that the majority of past studies focused on safety and structural integrity evaluations of seismic and fire loading. Only recently, a limited number of studies considered external blast and aircraft impact scenarios that included structural disaster prevention focusing on structural design. For critical infrastructure such as RCCV, disaster prevention and blast protection against extreme loading scenarios must be incorporated in the design stage. The damage or collapse of the structures can lead to insurmountable environmental problems and human casualties. Due to current worldwide concern about bomb related terrorism and accident, researches on minimizing structure and infrastructure damages from these events are actively pursed all over the world. However, due to restrictions of test facilities and cost, most researches are conducted as analytical formulation and computer simulation where reliability of the study results are questionable^7^^,^^9^^,^^11^.

Choi et al.^4^ conducted a simulation on the internal pressure buildup scenario of prestressed concrete containment vessels (PCCV), Moon et al.^7^ also analyzed the behavior of PCCV at high temperature and pressure buildup loading. Only a limited number of studies focused on the external blast and low-speed velocity impact loading to PCCV and RCCV^3,4,7,15^. The only meaningful study to date on internal loading of RCCV was performed at Sandia National Laboratory (SNL) in the United States (U.S.), in which experiments are conducted on 1/4 and 1/3 scale steel containment vessel and PCCV, respectively, by applying slow internal pressure buildup loading^8^. However, based on experiences of extreme disasters, the degree of structural damage was much more serious for the blast loading than the internal pressure buildup loading. However, due to cost and testing site restrictions, no meaningful studies of internal blasts were conduct on PCCV or RCCV. Therefore, in this study, internal blast loading test on RC tubular member was performed to obtain the data for the calibration of the internal blast applied RCCV analysis. The weight of the explosive charge was varied to understand the explosive pressure variation effect on RCCVs.

## Internal blast experiment details

### Internal blast characteristic

**Figure 1:**
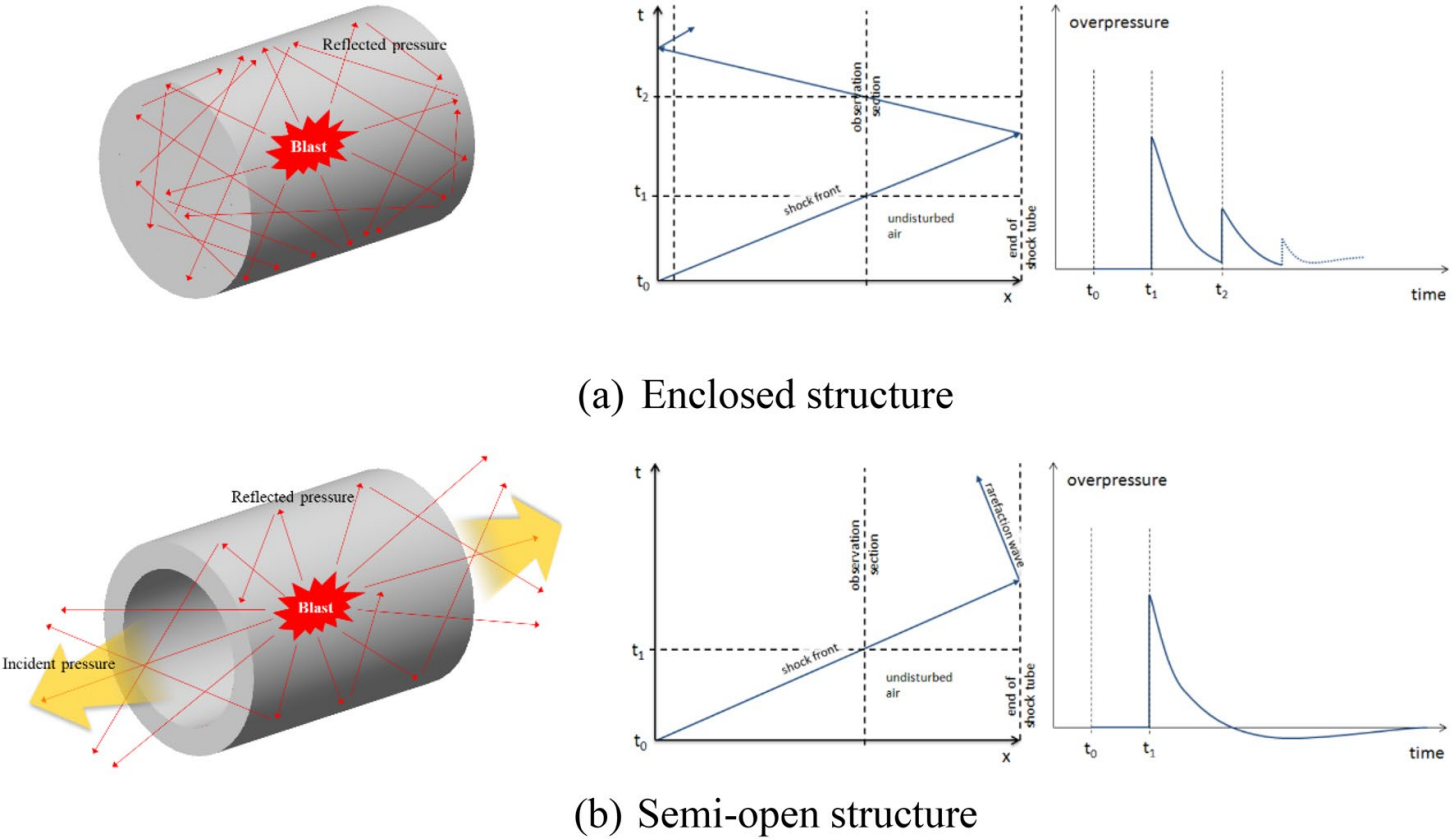
Schematic drawing of internal blast pressure propagation. (**a**) Enclosed structure. (**b**) Semi-open structure.

Blast loading is classified as either restrained or unrestrained blast, which can be considered as primarily external and internal explosion, respectively. Depending on the blast conditions, it is classified as (a) air blast without ground effect, (b) air blast with ground effect, or (c) enclosed space blast. Unlike an external explosion, a restrained blast generates a relatively large amount of pressure from the internal enclosed characteristic, generating greater blast pressure due to the conservation of blast energy inside a structure. The blast pressure interactions come from combined effect of shear wave, reflected wave, and gas pressure. As shown in Fig. 1, when a direct blast pressure reaches the inner wall surface of a tube structure, an interaction of free-field incident and reflected pressure due to bouncing phenomenon inside an enclosed space magnifies the resultant pressure. This pressure interaction continues until the pressure dissipates. It is important to note that the pressure magnitude magnifies to a maximum value, then slowly dissipates to zero pressure. In turn, the magnification of the pressure causes greater damage to the structure compared to the external blast loading without the interaction effect^5^. As shown in Fig. 1a, if an internal blast occurs in a fully enclosed RCCV, data acquisition is nearly impossible, due to reflecting blast pressures inside the structure destroying the pressure gauges and strain sensors attached to the inner surface. Therefore, in this study, the internal blast was detonated inside of a semi-open specimen as shown in Fig. 1b. The opening allowed a partial release of the internal blast pressure to control the pressure magnitude to be able to obtain pressure data. The blast pressures which were released to the left and right open ends of the specimen were measured by incident pressuremeters placed at a certain distance from the opening while the reflected pressure inside of the specimen was measured by a reflected pressuremeter attached to the inner.

Damage assessment of nuclear containment structures according to explosion loading scenarios have been partially verified in previous studies. Using the external blast loading scenario, the structural behavior of the walls of containment vessel at the Uljin Units 1 and 2 were analyzed. The scenario applied for experimental and analytical damage assessment of outer wall of containment vessel was an external blast charge, exploding at a charge distance of 1.0 m^1–3^. An internal blast scenario of a charge explosion due to unidentified explosive installation or mechanical device failure inside the containment vessel structure was used for this study. The average blast pressure (P_r_) and average unit impulse (i_r_/W^1/3^) were calculated based on TM5-1300 (UFC 3-340-02). The explosive pressure load was estimated from the data obtained from the experiment. Because the pressure was bouncing multiple times in the enclosed space, pressure considered in the analysis is based on only the pure initial blast pressure reaching the inner surface.

There are various types of explosives, but the typical explosives used in blast tests are TNT and ANFO explosives. TNT explosives can cause damage to specimens and measurement sensors due to the impact caused by the steel shell debris when an explosion occurs. Therefore, in this study, an ANFO explosive that does not require a steel shell was selected. The ANFO explosion creates 3300 m/s detonation speed with 145% capacity of the ballistic mortar. It was confirmed that energy is emitted mainly in the form of high-temperature and high-pressure gas. The shape of the ANFO charge is a ball type, so a ball shape ANFO charge was used in all tests.

However, the calculation for the blast pressure was only considered for external blast loading, where the bouncing effect of the blast pressure by the internal blast loading was not considered in UFC-3-340-02.

Therefore, for quantitative internal blast loading pressure measurement considering the bouncing effect within the specimen, it is necessary to derive an internal explosive charge weight that does not cause damage to the measurement sensor. Blast pre-tests were performed according to various explosive charge weight to determine the explosive charge weight that can induce specimen failure. In the main test, the explosive charge weight of 15.88, 20.41, 22.68 and 24.95 kg were used to consider the pressure variation effect.

### Test specimen details

Test specimens were modeled and designed based on a target structure of RCCV of Kori 1 and 2 NPP. The RCCV was designed as a RC structure with a service life of 40 years. The actual RCCV consisted of a tubular wall and an elliptical dome. For the RCCV wall, the reinforcement ratio was 0.024 and design concrete compressive strength was 41.37 MPa. The tubular specimens without the dome and lining plate were fabricated by scaling down the wall thickness while applying the same reinforcement ratio and target concrete compressive strength as the original structure.

Previous studies on blast loading researches on containment vessel for external blast and impact resistance were conducted by Choi et al.^3^. In their study, external blast and impact loading tests were performed on scaled down RC slabs based on the outer wall of a containment vessel. In this study, a semi-closed specimen behavior was analyzed when internal blast pressure was applied to the wall, excluding the blast pressure that escaped to the open space. Therefore, calibration data for internal blast pressure was obtained from internal blast test.

Although experimental studies on fully-enclosed structures should be performed, it is impossible to obtain meaningful data due to the catastrophic failure of the structure from its enclosed characteristic causing damages to the measurement sensors. Also, it is difficult to perform analytical and numerical evaluation, because it is impossible to secure calibration data from the blast test on a fully-enclosed structure.

The location most vulnerable to internal blast pressure is the central (mid-height) location of the RCCV wall. It is important to note that this type of internal blast test was never attempted previously. Four RC specimens were blast tested using ANFO charge of 15.88, 20.41, 22.68, and 24.95 kg, which were titled as RC35, RC45, RC50, and RC55, respectively.

**Figure 2:**
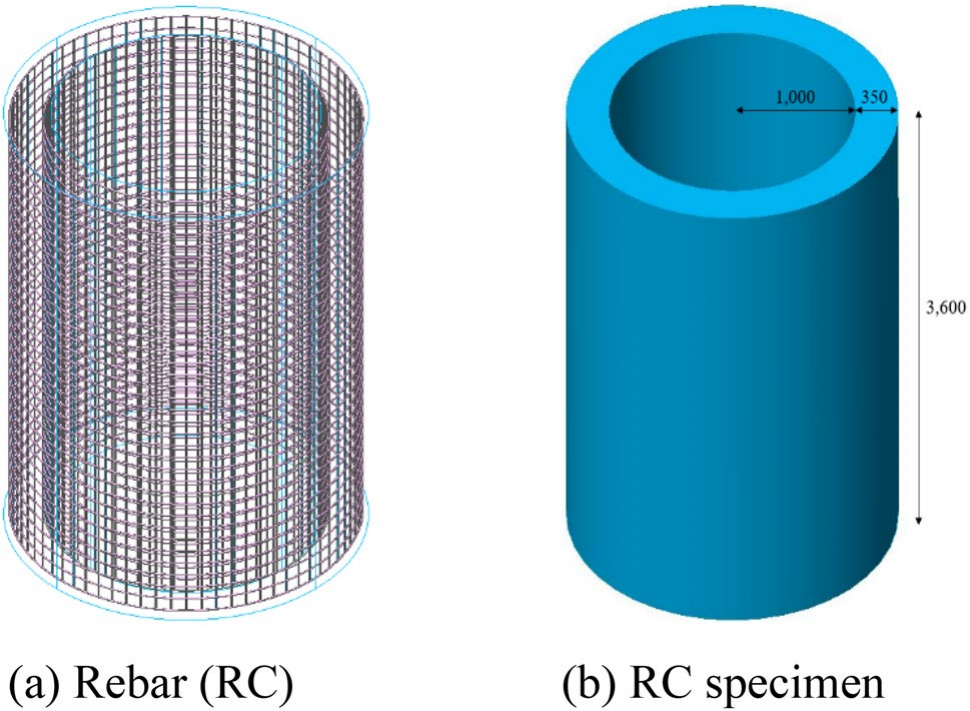
Rebar and specimen details (unit : mm). (**a**) Rebar (RC). (**b**) RC specimen.

The RCCV scale-down model was selected so the test is manageable and conductible based on the size of the test site and the blast pressure. Therefore, the specimen was selected as a 1/20 scale-down model according to the outer diameter and height of the real scale RCCV. Outer and inner diameter of the RC tubular specimens was 2700 mm and 2000 mm, respectively, as shown in Fig. 2. The wall thickness was 350 mm and the longitudinal tube length was 3600 mm. The tube thickness was designed with a required minimum concrete cover thickness of 50 mm and the reinforcement ratio of 0.024, as same as the real scale RCCV wall. A RCCV is normally designed to have a 6 mm thick steel liner plate to prevent radiation leakage in case of malfunction of a nuclear reactor. However, in this study, the steel lining acts as a shielding containment against radiation leakage and does not affect the structural resistance to internal blast or pressure. The structural resistance of RCCV concrete containment vessel under the internal pressure comes from the RC wall itself. Therefore, steel lining was not implemented in the study. As shown in Fig. 2, D13 reinforcement were arranged in a grid configuration with a spacing of 100 mm and a unit weight of 1101 kg/m. The specimens were cast using concrete with 28-day compressive strength of 40 MPa. The mechanical properties of concrete and rebar of test specimen were shown in Table 1.

**Table 1:** Mechanical properties of the concrete and steel rebar used in the tests.

Type		
Concrete	Compressive strength (MPa)	40.00
	Elastic modulus (GPa)	30.46
	Poisson's ratio	0.17
Rebar	Yield strength (MPa)	413.68
	Tensile strength (MPa)	620.53
	Elongation (%)	7.00

### High strain rate compatible measurement system

**Figure 3:**
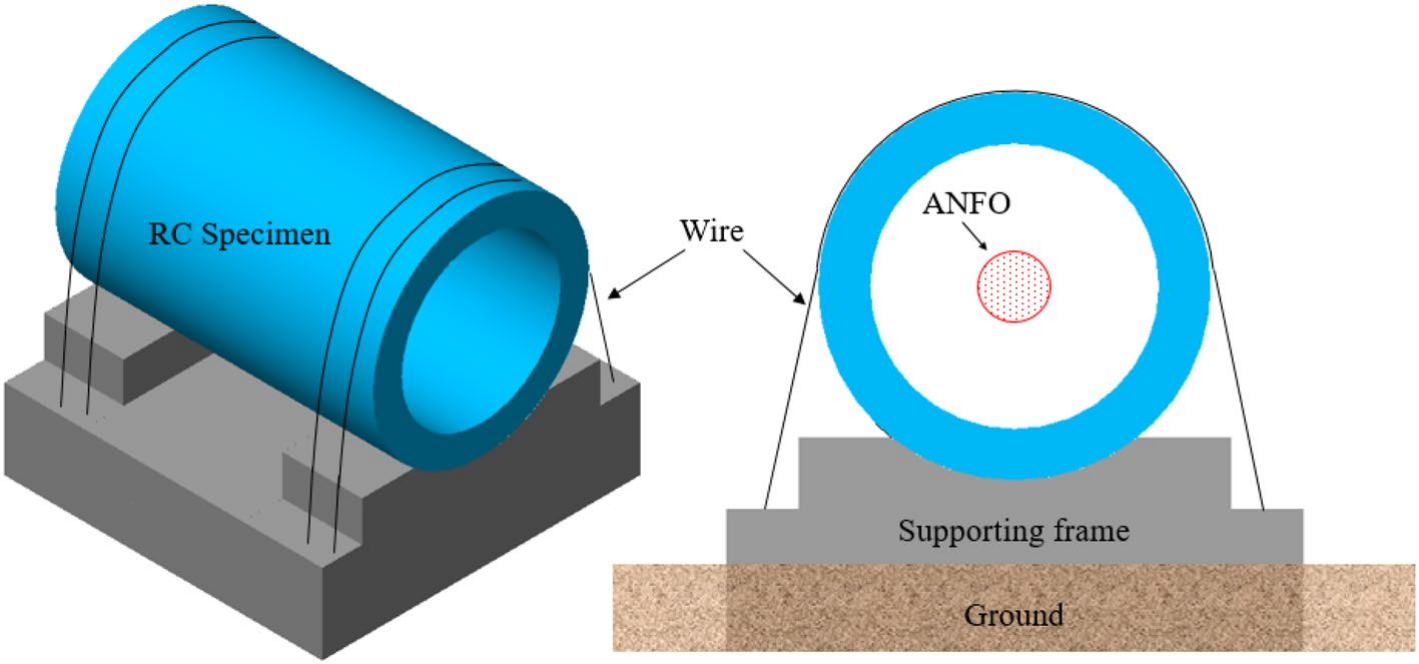
Details of supporting frame.

The blast test was conducted at Darakdae Test Site operated by the Agency for Defense Development (ADD) Research Center in Korea. As shown in Fig. 3, a frame structure with a clearance of 1000 mm from the ground surface was used to support the specimen. The tubular specimen with a weight of 2600 kg was mounted on the support frame and tightened at both ends using 100 mm sling wire, chain block, and fastening buckle to maintain full contact between the specimen and the support frame throughout the test. A 10 mm thick rubber pad was inserted between the specimen and the support frame to prevent damage to the specimen and to minimize the specimen’s movement during internal blast loading. The setup for the specimen test is schematically shown in Fig. 3. Strain gauges for rebar and concrete were also embedded in the support frame to measure the strain on the support frame from internal blast loading. The ANFO was placed at the center of the cross-section at the mid-span using four ties installed at 90° intervals to secure the explosive charge in position.

**Figure 4:**
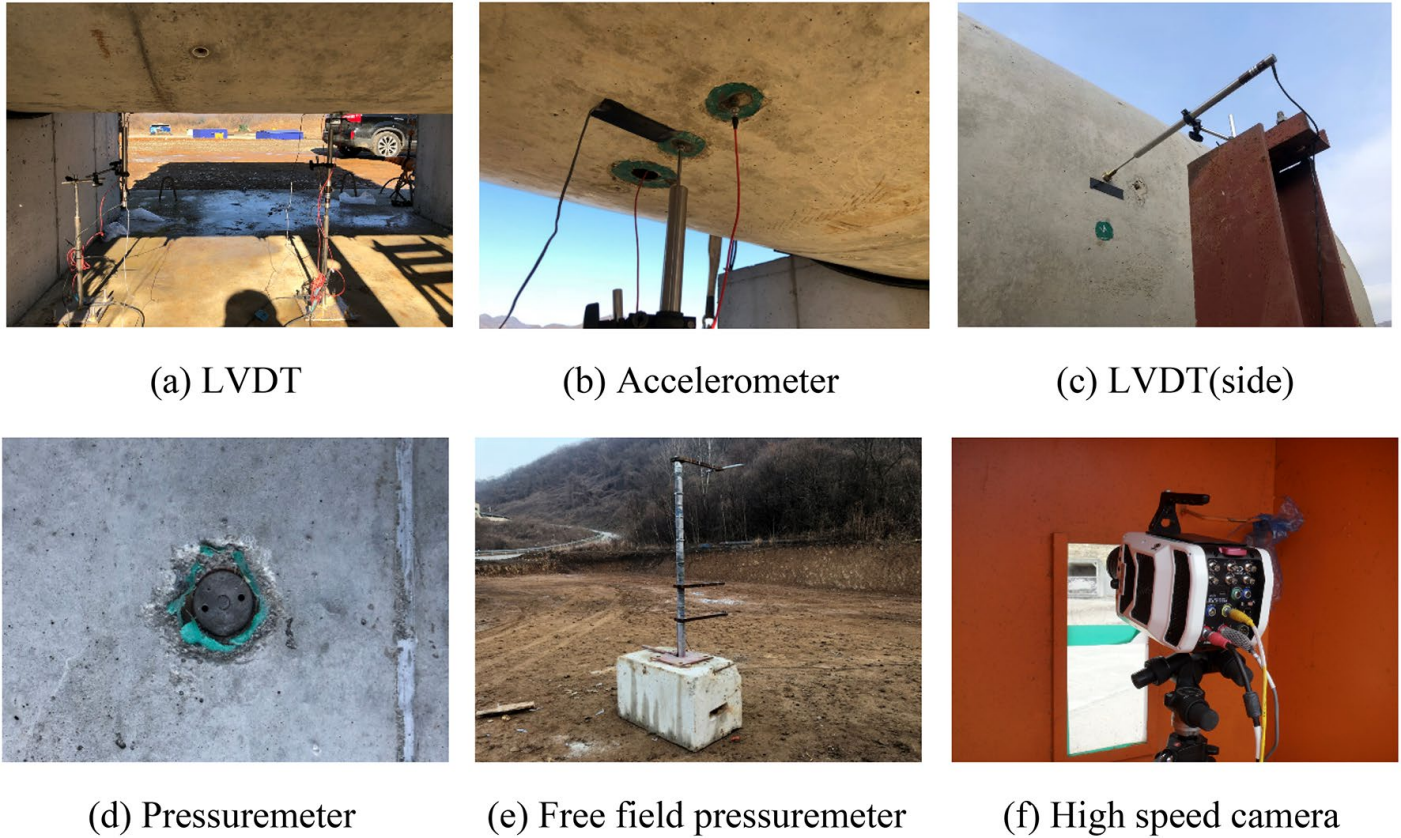
Measurement equipment. (**a**) LVDT. (**b**) Accelerometer. (**c**) LVDT(side). Figure 4(**d**) Pressuremeter. (**e**) Free field pressuremeter. (**f**) High speed camera.

**Figure 5:**
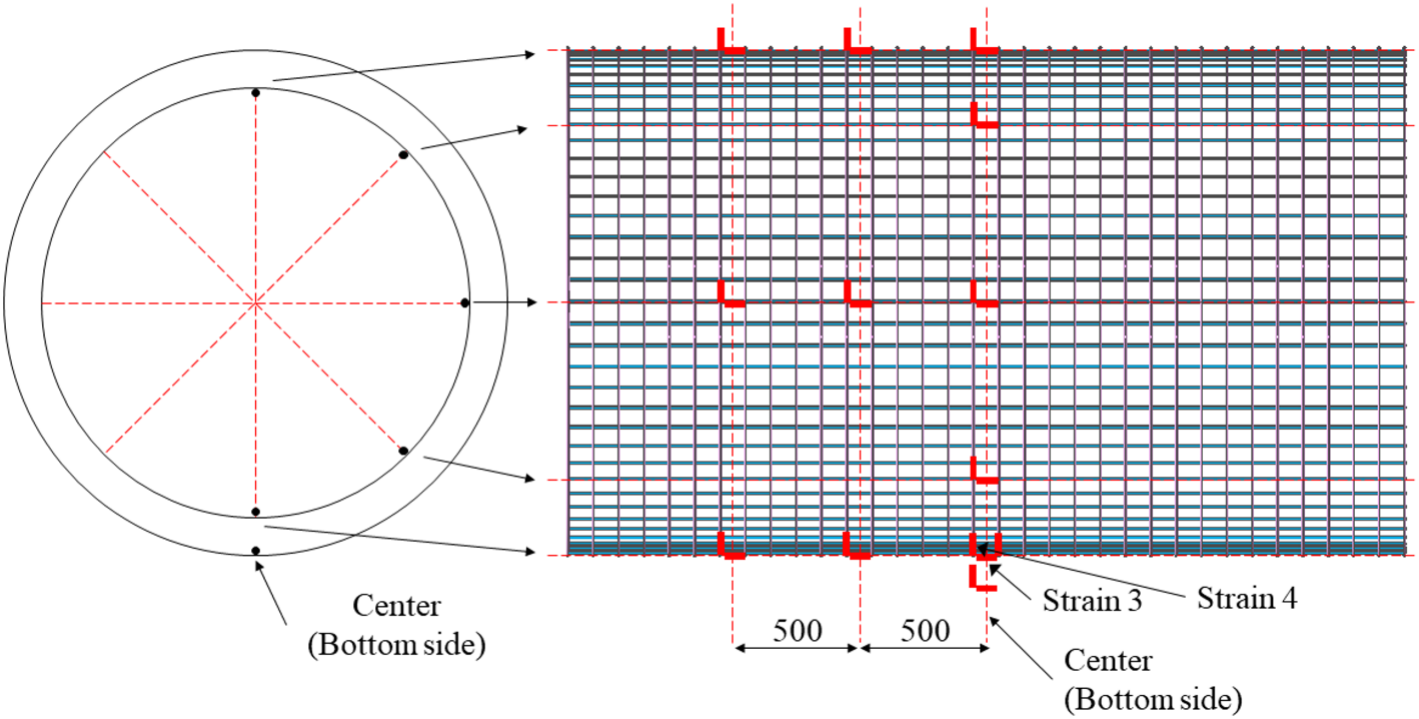
Strain gauge locations.

Precise measurement of the behavior of the specimen in response to the internal blast require instantaneous measuring equipment and data acquisition systems. Figure 4 shows the photos of linear voltage displacement transformers (LVDTs), accelerometer, pressuremeters, and high-speed camera used for the measurement. The locations of the embedded rebar gauges are shown in Fig. 5.

**Figure 6:**
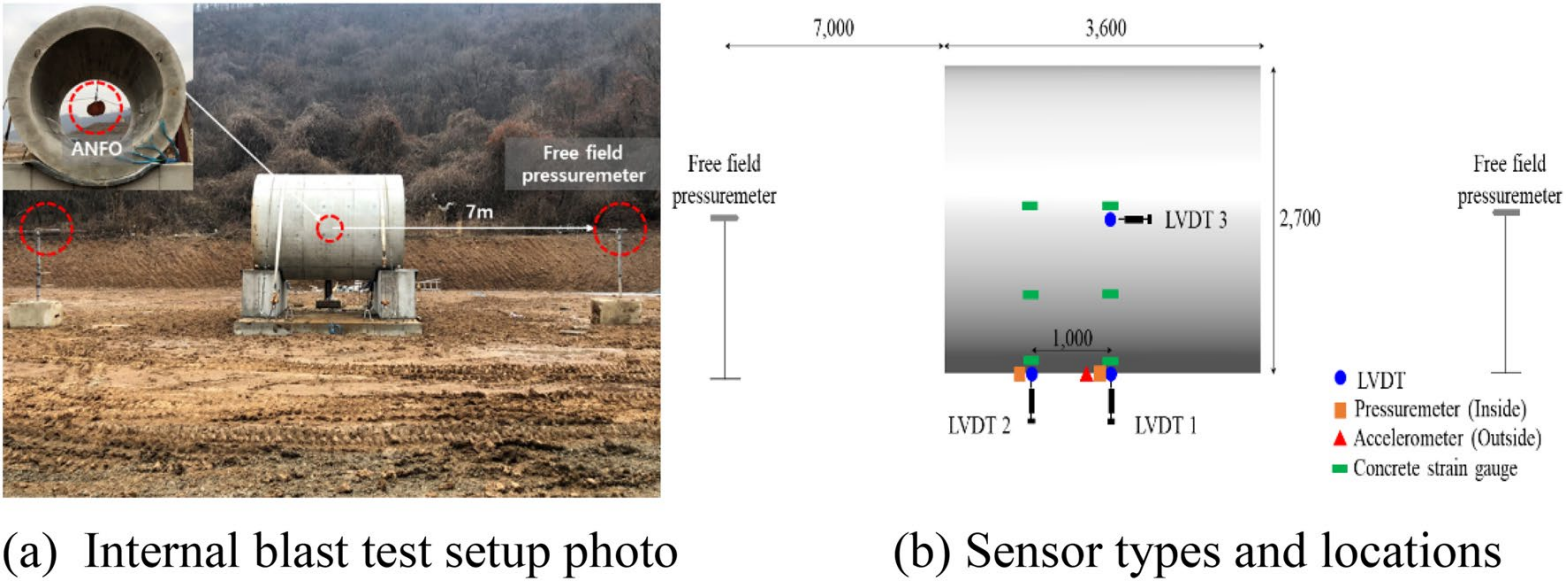
Test setup and measurement details. (**a**) Internal blast test setup photo. (**b**) Sensor types and locations.

The sensors were attached and buried with jigs during fabrication of the specimens to ensure full attachment to minimize measurement errors during the blast. The free-field incident pressure of the blast loading was measured using two 3447 kPa capacity instant pressuremeter installed at 7000 mm from the left and right end openings of the specimen at the same height as the blast charge. The reflected pressure of the internal blast was measured using two reflected pressuremeters attached to the internal surface of the tubular specimen at the mid-span and 1000 mm from the mid-span. The maximum and residual deflections were measured from the exterior surface at the mid-span and 1000 mm from the mid-span using ± 100 mm spring type dynamic LVDTs. An accelerometer with an allowable range of 50,000 g was used on the external surface at the mid-span, which was the same location as a mid-span deflection measuring LVDT. In total, six concrete gauges were embedded at 1000 mm from the mid-span of the specimen at 45° intervals. Nineteen rebar gauges were attached in the inner and outer rebar at the mid-span of the specimen in 45° intervals in both longitudinal and lateral directions. The details of the measurement gauge locations and types used in the test are shown in Fig. 6.

To ensure safety of the researchers, data acquisition was conducted in a test control room located approximately 50,000 mm from the test site. The data loggers used for data acquisition were Dewetron 1201 and Dewetron 2600 that could sample signals at 200–500 kHz. Pressure, acceleration, and deflection were measured at a rate of 500 kHz and the strains in the rebar and concrete were measured at a rate of 200 kHz. For visual inspection of the internal blast pressure, video images of blast were recorded using a 4000-frame per second high-speed camera. Figure 6 shows the equipment and sensors used in the test.

## Internal blast test results

Free field pressure, deflection, strain, and environmental condition data for RC35, RC45, RC50, and RC55 are tabulated in Table 2. As shown in the table, when the blast charge weight increased, the magnitude of all of the data increased. For example, when the weight of explosive charge increased from 15.88 to 24.95 kg, the peak incident pressure and deflection stabilization time duration increased from 0.1718 to 0.3394 MPa and from 5.856 to 5.981 ms, respectively.

**Table 2:** Summary of test results under internal blast loading.

Value		RC35	RC45	RC50	RC55
**Free field pressure**					
Peak pressure (MPa)		0.172	0.297	0.317	0.339
Duration (msec)		5.981	5.856	5.826	5.881
Impulse (Mpa-msec)		0.360	0.379	0.387	0.444
**Deflection (mm)**					
Maximum	Mid-span (0°)	6.57	14.67	15.27	16.25
	Mid-span (90°)	3.95	7.39	8.76	11.29
	1000 mm	5.58	8.13	8.37	8.64
Residual (Mid-span 0°)		2.87	7.02	7.84	8.44
**Strain **($\mu \varepsilon )$					
Rebar longitudinal	Maximum	536.84	908.24	1476.31	1487.70
	Residual	228.31	57.24	228.23	641.07
Rebar lateral	Maximum	3134.85	16,419.32	20,986.06	21,897.05
	Residual	153.47	6602.94	4813.75	11,941.37
Concrete	Maximum	59.75	169.22	186.57	760.17
	Residual	17.41	63.49	104.31	72.27
**Environmental condition**					
Temperature (°C)		9.2	6.3	2.9	− 6.0
Rel Humidity (%)		45	41	16	31

### Incident and reflected blast pressure

**Figure 7:**
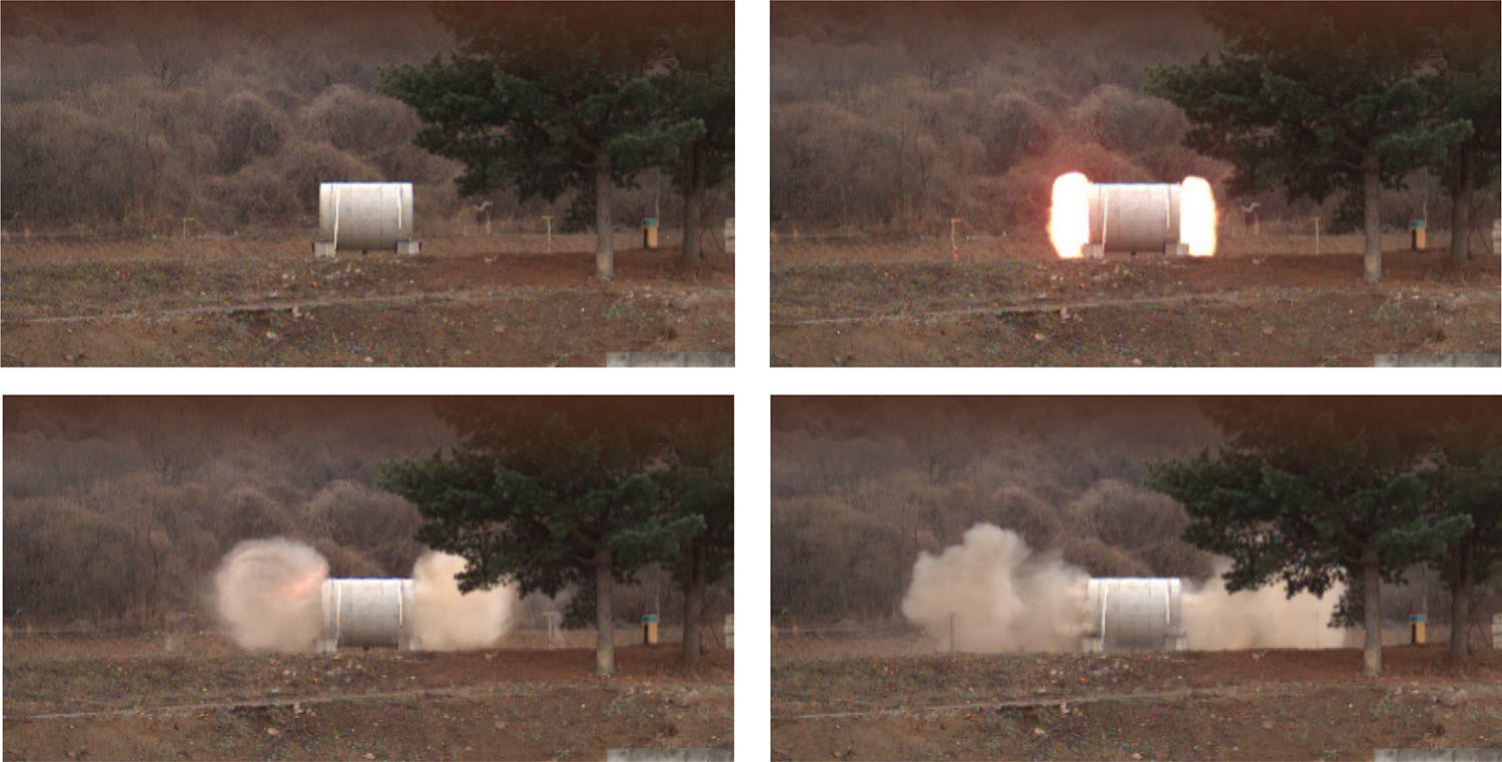
Energy release photos of ANFO 15.88 kg.

**Figure 8:**
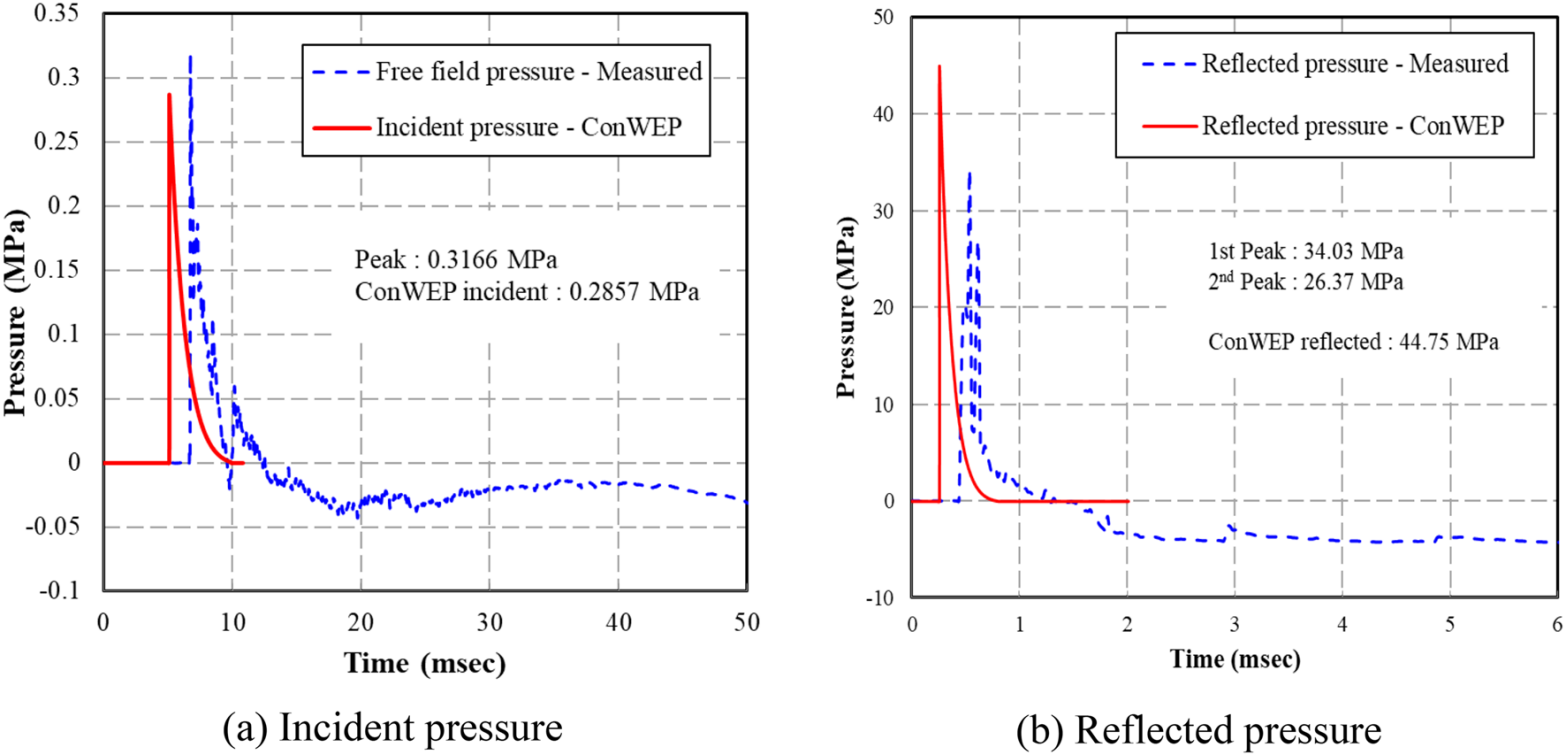
Blast pressure results of ANFO 15.88 kg. (**a**) Incident pressure. (**b**) Reflected pressure.

Photos from the high-speed camera taken from the internal blast test are shown in Fig. 7. The figure shows that ANFO blast pressure was released to the left and right openings of the specimen in the form of high temperature and pressure gas. As shown in Fig. 8, the pressure data obtained from RC50 is compared to the calculated results from ConWEP program, a program to estimate blast pressure based on UFC3-340-1^12^. In order to filter a large amount of pure pressure data measured with a pressuremeter, frequency and magnitude were systematically selected through the advice of a data analytics professional. Finally, the time-pressure curves shown in Fig. 8 according to the time interval of 0.004 ms are drawn. Figure 8 shows the free-field incident and internally reflected pressure in relation to the time of the ANFO 22.68 kg charge detonation measured from the pressuremeter at a distance of 7000 mm from the mid-span. For RC50, the measured peak measured pressure was 0.3166 MPa and the impulse was 0.4717 MPa-msec. ConWEP calculated incident peak pressure was 0.2857 MPa and the impulse magnitude was 0.2946 MPa-msec. The trend of ConWEP calculated incident pressure was similar to the test pressure. However, ConWEP calculated impulse pressure was 41.58% lower than RC50 test data. As shown in Fig. 8b, the measured reflected pressure of RC50 was approximately 10 MPa lower than ConWEP calculation. The test results also showed that there was a second peak reflected pressure after 1 ms, indicating that the pressure reflection occurred inside the specimen. As shown in Fig. 8a,b, the time to reach the peak incident and reflected pressure calculated from ConWEP was approximately 0.3 ms faster than the measured time from the test. The difference between the measured and calculated results is likely due to ConWEP being an external blast pressure calculating program, which is unable to consider internal reflections and interactions of various types of the internal blast pressures.

### Time-deflection relations

**Figure 9:**
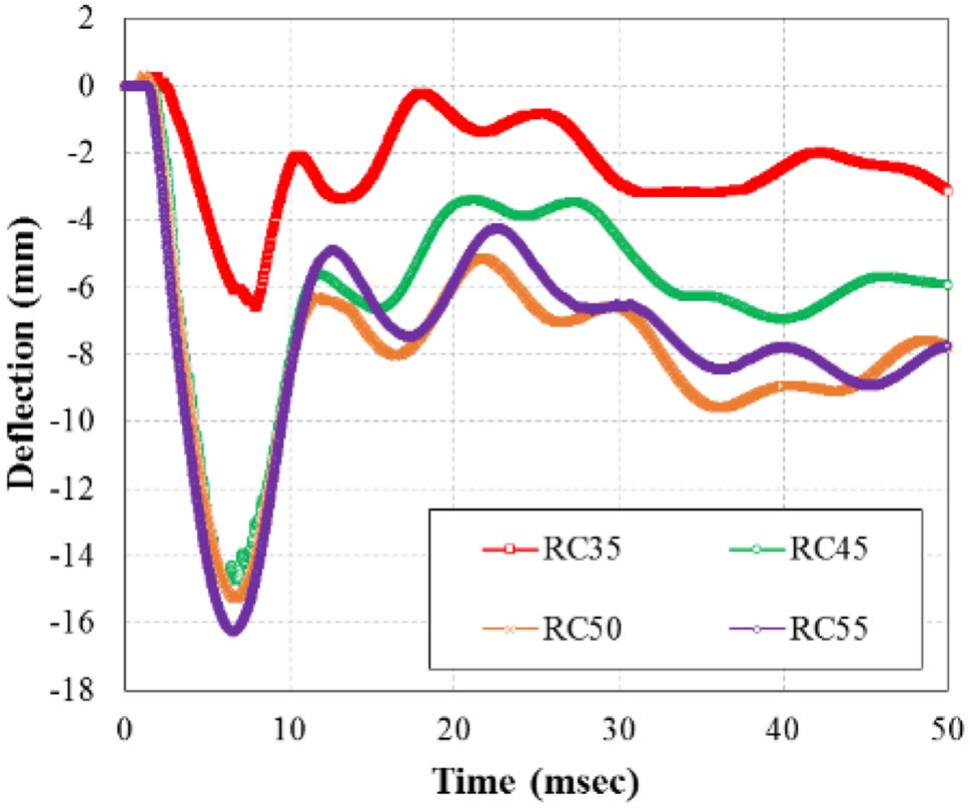
Time-deflection curves of specimens.

Deflection data of the RC specimens were measured from the dynamic LVDTs installed at three locations on the external surface at mid-span 0° and 90° as well as 1000 mm from the mid-span 0°. For RC50, the maximum and residual deflection at the mid-span was 15.27 and 6.62 mm, respectively. In RC50, the deflection behavior was a cyclic type due to repeated application of reflected pressures to the interior surface of the specimen. As shown in Fig. 9, plastic deflection occurred in RC50 due to the damage of the wall from the initial direct blast pressure.

**Figure 10:**
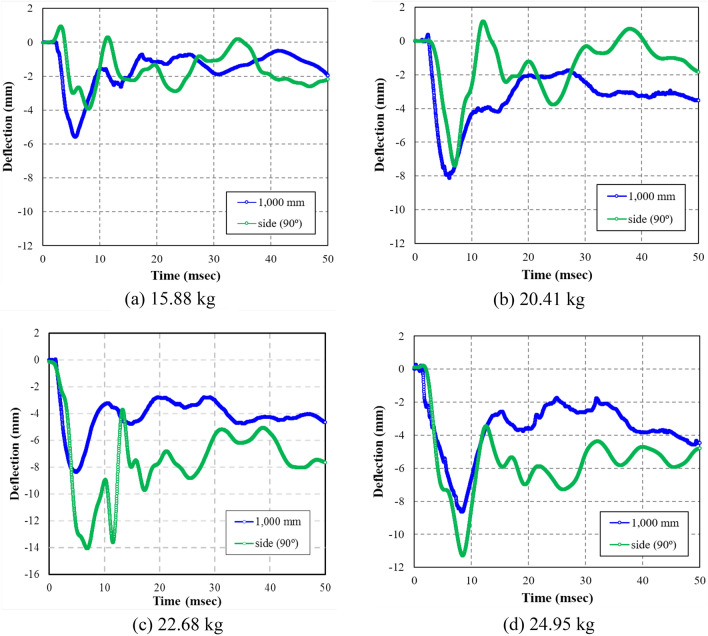
Time-deflection curves of LVDT 2 (1000 mm) and LVDT 3 (side 90°). (**a**) 15.88 kg. (**b**) 20.41 kg. (**c**) 22.68 kg. (**d**) 24.95 kg.

The maximum deflection for RC35, RC45, RC50, and RC55 was 6.57, 14.67, 15.27, and 16.25 mm, respectively. Compared to the maximum deflection of RC35, the maximum deflection of RC45, RC50, and RC55 was 223.28, 232.42, and 247.34%, respectively, to the maximum deflection of RC35. The residual deflection of RC35, RC45, RC50, and RC55 was 2.87, 7.02, 7.84, and 8.44 mm, respectively. The results indicated that RC35 had a much smaller residual deflection than the other specimens. Based on the residual deflection results, it is safe to assume that RC35 behaved primarily in an elastic manner with minor plastic deflection, while other specimens were catastrophically damaged by the blast, resulting in large residual deflections. In addition, as shown in Fig. 10, the LVDT results at RC35 and RC45 specimens showed greater mid-span deflection at the bottom surface (at the mid-span 0°) and 1000 mm away from the mid-span (0°) than at the side surface (90°), which can be attributed to the effect of gravity when the internal blast pressure originated at the center of the mid-span. However, the LVDT displacement results at RC50 and RC55 specimens were larger at the side surface (90°) than 1000 mm away from the mid-span (0°), which was induced by the intensive pressure applied to the mid-span due to the explosive pressure overcoming effect of gravity.

### Rebar and concrete strains

**Figure 11:**
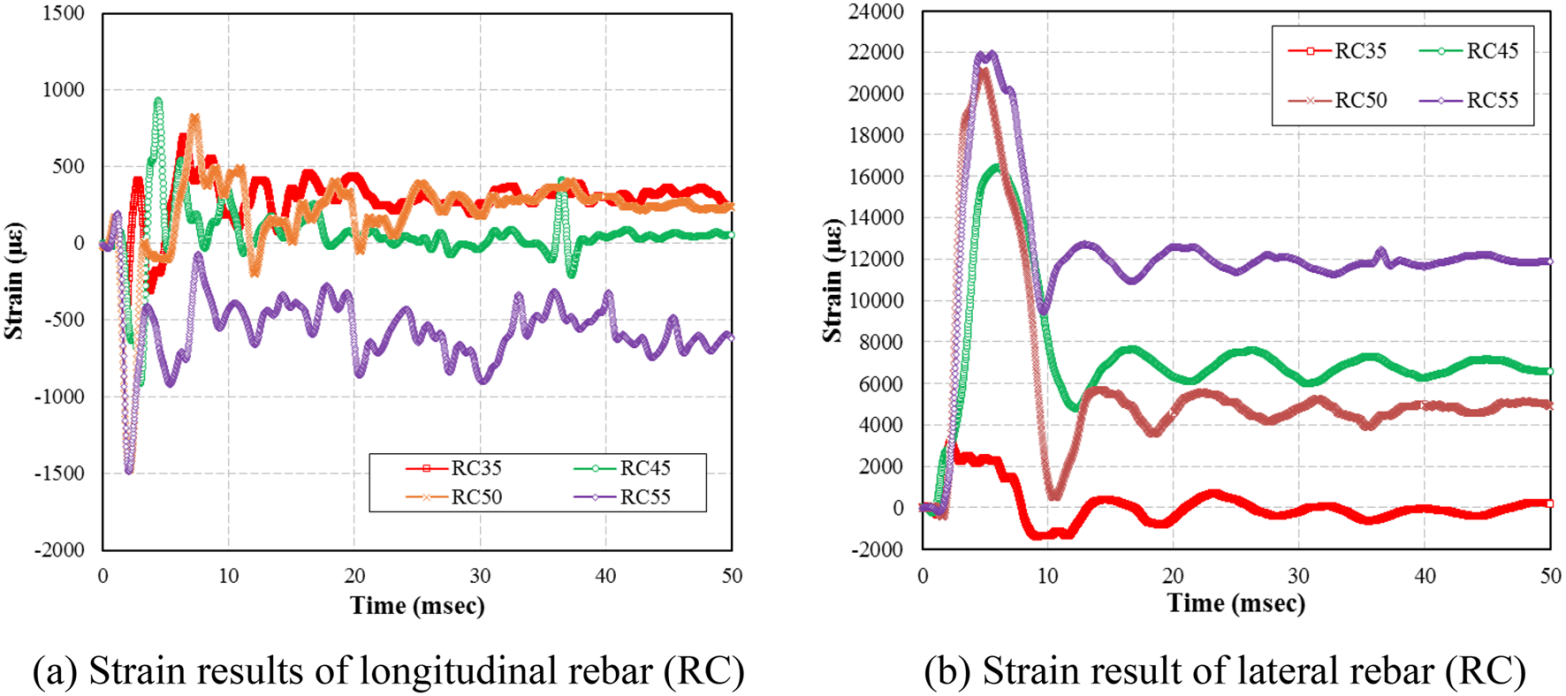
Steel strain results. (**a**) Strain results of longitudinal rebar (RC). (**b**) Strain result of lateral rebar (RC).

Strain data for the RC specimens for various ANFO explosive charge weights are shown in Fig. 11a. The maximum strain for the longitudinal rebar of RC35, RC45, RC45, and RC55 was 536.84 με, 908.24 με, 1476.31 με, and 1487.70 με, respectively. The maximum strain on RC45, RC50 and RC55 was 169%, 275%, and 277%, respectively, to RC35 maximum strain. The residual strain on the rebars of RC35, RC45, RC45, and RC55 was 228.31 με, 57.24 με, 228.23 με, and 641.07 με, respectively. Unlike the maximum strain data, there is no clear trend shown from the residual strain data. The apparent random trend can be attributed to the fact that RC35 was behaving mostly in elastic manner while the other specimens catastrophically failed at the application of the initial direct blast pressure. This failure behaviour can be verified by the crack patterns on the specimens, which will be discussed in detail in “Crack patterns”section. As shown in Fig. 11b, the maximum strain on the lateral rebar of RC35, RC45, RC50, and RC55 was 3134.85 με, 16,419.42 με, 20,986.06 με, and 21,897.05 με, respectively. The maximum strain on RC45, RC50, and RC55 was equivalent to an increase of 424%, 569%, and 599%, respectively, to the maximum strain of RC35. These results suggested that both longitudinal and lateral rebar strains at the mid-span of the specimens increased in proportion to the magnitude of the internal blast pressure.

As shown in Fig. 11a,b, the lateral rebar strains are much larger than the longitudinal rebar strains, due to the rebar layout arrangement. In the RC specimens, the lateral rebars wrapped around the longitudinal rebars, which makes the longitudinal rebar movement to be confined by the lateral rebars. Since the longitudinal rebar movements were restricted whereas the lateral rebars were allowed to move freely, this rebar layout would induce larger lateral rebar strains than the longitudinal rebar strains.

**Figure 12:**
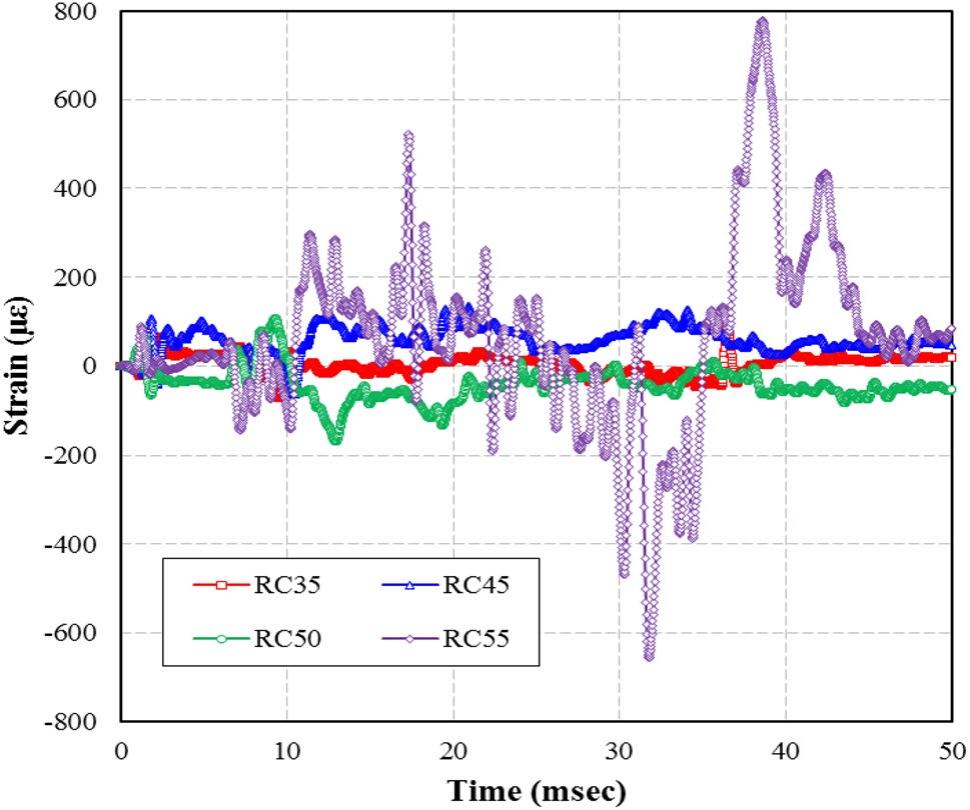
Concrete strain results.

As shown in Fig. 12, the maximum strain on the concrete in RC35, RC45, and RC55 was 59.75 με, 169.22 με, and 760.17 με, respectively. For RC45 and RC55, these represent an increase of 283%, and 1272%, respectively, to the strain of RC35. The maximum rebar strain occurred within 10 ms from the initiation of the blast. However, the maximum strain on the concrete occurred within 50 ms due to continuous crack propagations caused by the reflected blast pressure. Also, as the weight of the blast increased, the concrete strain was relatively small in RC35, RC45, and RC50, while much larger in RC55. The significant difference in crack pattern between the specimens showed that RC55 was damaged to the point of being unable to bear any load. These behaviors were verified by the crack patterns in the specimens.

### Crack patterns

**Figure 13:**
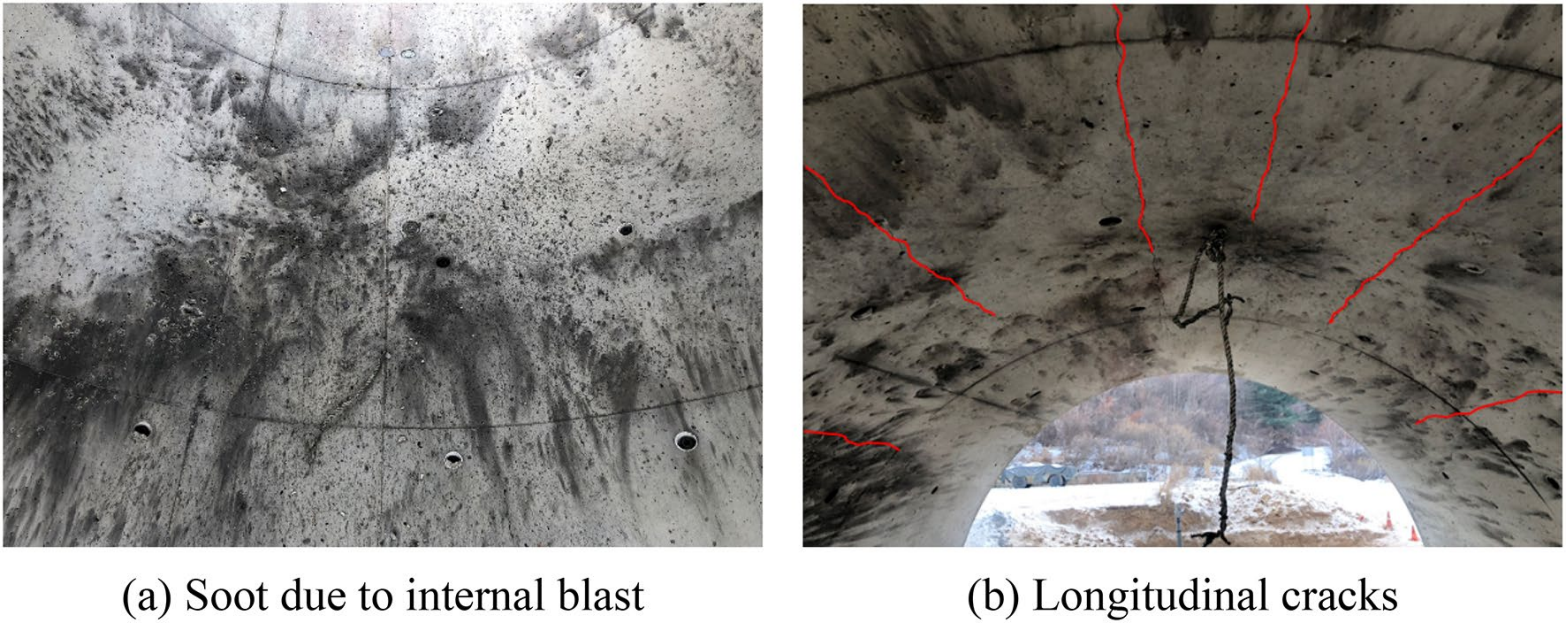
Crack patterns of specimens. (**a**) Soot due to internal blast. (**b**) Longitudinal cracks.

Crack patterns were visually examined to determine the extent of damage to the specimens. Interior cracks were examined while the specimen was on the support frame. The specimen was then carefully lifted perpendicularly to a standing position in order to examine the exterior cracks. For RC50 specimen, there was not shown observable internal damage from spalling as shown in Fig. 13, because the internal pressure generated mostly compressive stresses which were resisted by rebars. The crack pattern on the interior surface of the specimen progressed in a longitudinal direction (e.g., the direction of the longitudinal rebar) as shown in Fig. 13b. However, the crack width was relatively small.

**Figure 14:**
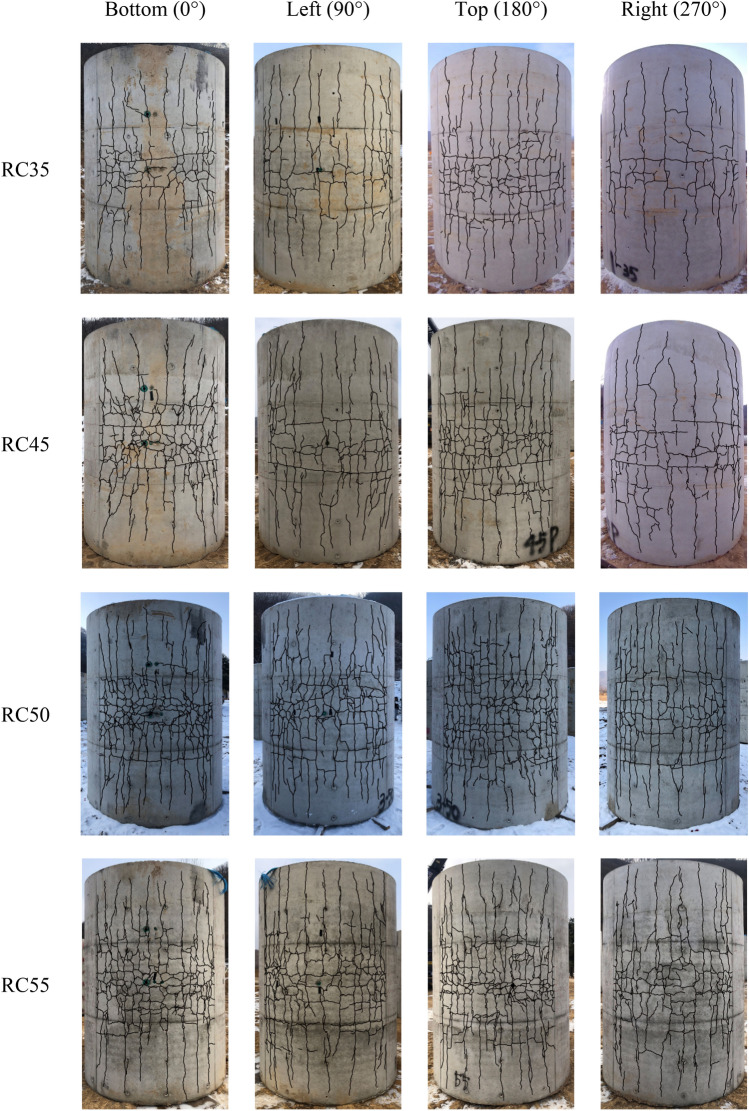
Specimen crack patterns.

Crack patterns of the exterior surface of the specimen are shown in Fig. 14. As shown in the figure, the exterior surface had more cracks in both longitudinal and lateral directions with much wider crack width than the interior surface. Also, as shown in the crack pattern in Fig. 14, it can be seen that the internal blast load pressure is concentrated in the center of the internal wall. Due to the pressure escaping through end openings, the pressure distribution along the axis does not occur significantly. The longitudinal cracks were evenly distributed around the specimen where the cracks formed between the longitudinal rebar. However, similar to the lateral crack pattern, the longitudinal cracks were also concentrated at the mid-span. As expected, the number of longitudinal cracks increased as the weight of the charge increased. The lateral and longitudinal crack patterns can be explained based on the rebar strain data. Because the lateral rebar strain was much greater than the longitudinal rebar strain, the concentration of lateral cracks was more significant than the longitudinal cracks at mid-span. This was due to the lateral rebar strain exceeding the longitudinal rebar strain at the mid-span.

### Acceleration

**Figure 15:**
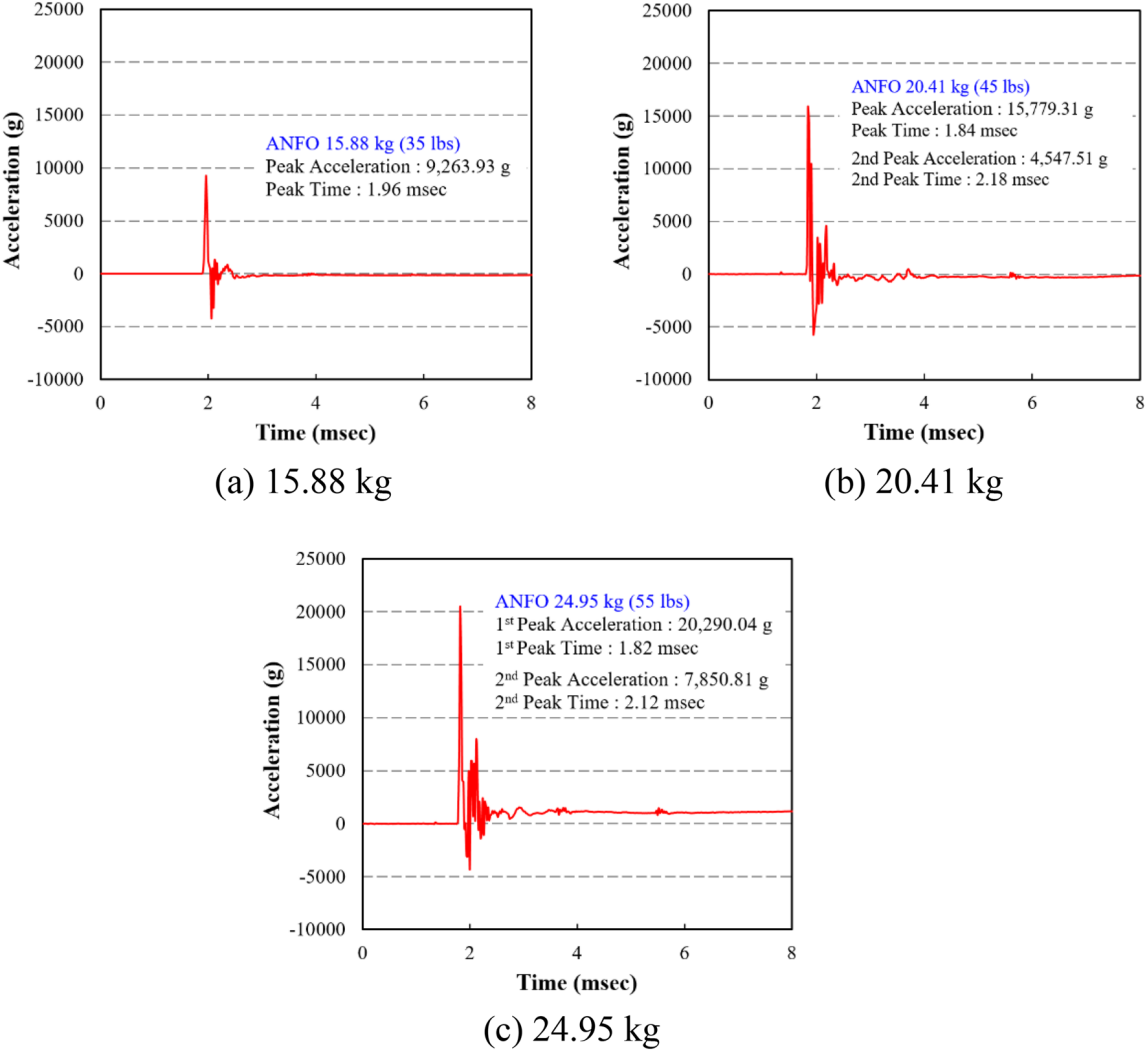
Acceleration results. (**a**) 15.88 kg. (**b**) 20.41 kg. (**c**) 24.95 kg.

Accelerated vibration of the specimen wall under internal blast loading was measured by accelerometer. Using the acceleration data obtained from the test, the dynamic increasing factor (DIF) could be obtained. As shown in Fig. 15, the maximum accelerations for RC35, RC45, and RC55 was 9263.93 g, 15,779.31 g, and 20,290.04 g, respectively. There was no acceleration data for RC50, because the accelerometer was destroyed by the blast pressure. Compared to the maximum acceleration of RC35, the maximum acceleration of RC45 and RC55 increased by 70.33% and 119.02%, respectively. The acceleration versus time plots of RC35, RC45, RC50, and RC55 are shown in Fig. 15. As shown in the figure, the overall acceleration trends were similar in all specimens except that the peak acceleration increased as the weight of the charge increased. Unlike RC35, the other two specimens experienced a significant second peak acceleration following the first peak acceleration, indicating significantly more reflection of the blast pressure occurred in those specimens. It is important to note that the time it took for the acceleration of RC55 to go to zero required much more time than in other specimens. More specifically, in RC55, it took approximately 250 ms for the acceleration to go to zero, whereas the acceleration of the other specimens went to zero immediately after the second peak acceleration. The time difference was attributed to the catastrophic damage in RC55 in which the specimen did not have sufficient stiffness in the wall to behave as a rigid wall.

## Result discussion and analysis

### RCCV Simple model

As shown in Fig. 9, the maximum deflection was much larger in RC45 than RC35. It is safe to conclude that the specimen subjected to an internal blast charge weight exceeding 15.88 kg caused a structural tensile failure, in which the specimen could not resist the load and induced plastic deformation. This conclusion is supported by the crack patterns discussed in “Crack patterns” section. In the RC45, RC50, and RC55 specimens, radial cracks in which lateral and longitudinal cracks were simultaneously observed in the central region. However, in the RC35 specimen, the lateral cracks were relatively distinct compared to the longitudinal cracks. Based on the observation, the following equations can be derived.

The correction factor $\left( {{\upgamma } = \frac{1}{\alpha }} \right)$ of an internal blast compared to an external blast can be expressed by Eq. (1) through a maximum internal blast force $\left( {F_{max} } \right)$, a wall stiffness (*K*) of the tube structure, and a wall deflection (*U*_*max*_).

1 $$ F_{max} = {\upalpha }\left( {K \cdot U_{max} } \right){ } $$

where *K* = *K*_*el*_ + *K*_*pl*_ and *U*_*max*_ = *U*_*el*_ + *U*_*pl*_ with the subscript *el* and *pl* denoting elastic and plastic, respectively. It is important to note that ${\upgamma }$ value has to be greater than 1.0, since an internal blast creates larger pressure magnitude due to the reflection effect of enclosed space compared to an external blast. The maximum applied force and deflection is compared for both elastic and plastic behaviors. If *K* and *U*_*max*_ are substituted into Eq. (1), then the equation becomes as follows.

2 $$ {\upgamma }F_{max} = \left( {K_{el} \cdot U_{el} { } + { }K_{pl} \cdot U_{pl} } \right) $$

**Figure 16:**
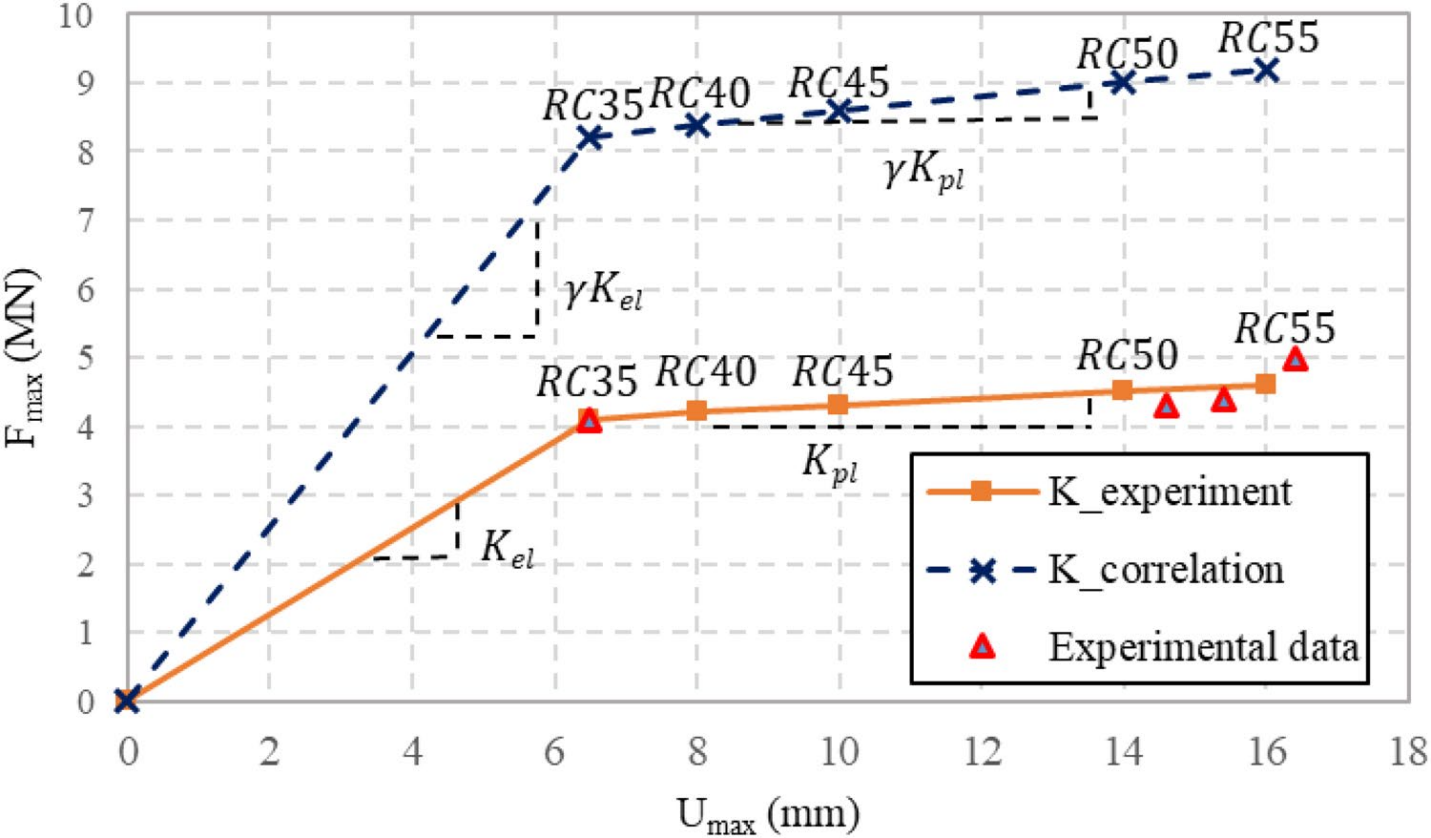
Measured elastic and plastic structural stiffness coefficient of the RC specimens.

Normally, it is nearly impossible to calculate or measure the structural stiffness coefficients for RC members under blast loading. However, in this study, because the pressure and deflection of the RC tubular specimens were measured from the test, *K*_*el*_ and *K*_*pl*_ could be obtained from the regression plot of *F* versus *U* test data as shown in Fig. 16. From the figure, a drastic and distinct change of slope of the curve is observed. Between RC35 and RC55, the stiffness changed due to residual plastic deflection.

**Figure 17:**
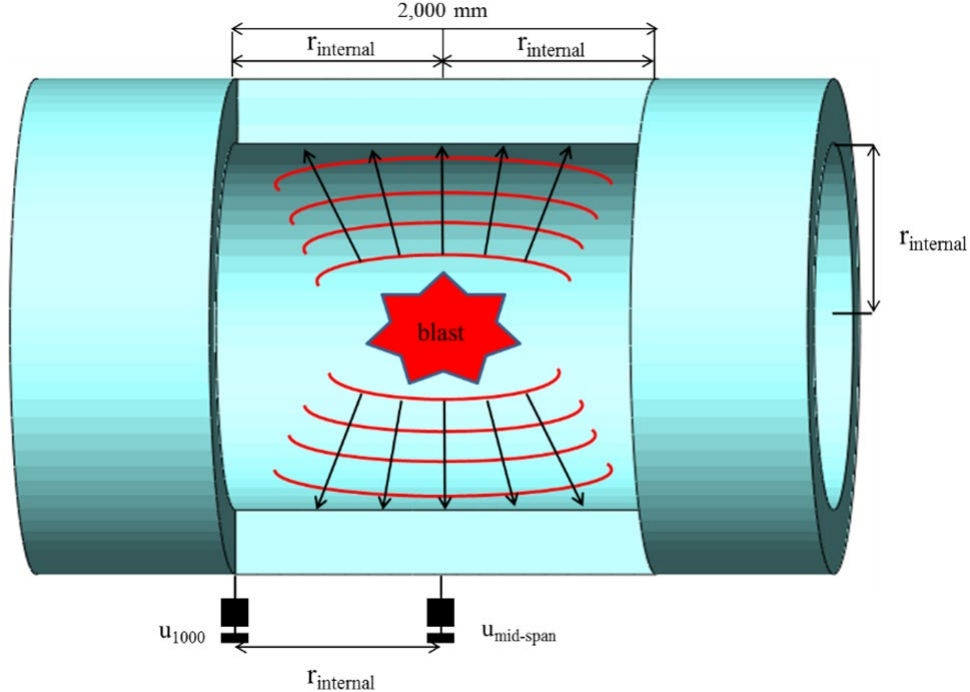
Internal blast simple model.

As shown in Fig. 17, it is assumed that the majority of the internal blast pressure was applied primarily to the left and right of the mid-span equaling a distance of 2*r*_*internal*_, equivalent to 2000 mm for this test. The assumption of the blast pressure distribution applied to the 2r_internal_ range of the simple model was performed for a conservative analysis. Unfortunately, there is no verification of accuracy available, but it is considered appropriate for a safety analysis of the structure.

As shown in Fig. 17, the mid-span deflection *U*_*mid-span*_ and the deflection of r_*internal*_ (= 1000 mm) from the mid-span *U*_1000_ were measured from the dynamic LVDTs installed at three locations on the external surface of the tubular specimen. If the majority of the pressure was applied between ± *r*_*internal*_ from the mid-span, then the maximum internal blast load *F*_*max*_ could be calculated by multiplying the internal surface area of the tube region that was applied with a majority of the blast pressure *P*_*max*_ to the pressure data obtained from the pressuremeters attached to the internal tube surface as shown in Eq. (3).

3 $$ F_{max} = 2\pi r_{internal} \cdot 2 \cdot r_{internal} \cdot P_{max} $$

The correction factor for the pressure ${\gamma  }$ of the internal blast loading can be calculated by calculating ${\upalpha }$ by inputting the initial peak pressure values in Eq. (3) to obtain $F_{max}$, which is then inputted into Eq. (2) with the values of $K_{el} , K_{pl} , u_{el}$, and $u_{pl}$ to obtain ${\gamma  }$ value. Then, ${\upgamma }$ is multiplied to $F_{max}$ to reflect the increase in the failure load data of the RC tubular specimens. The correction factor of ${\upgamma }_{35}$, ${\upgamma }_{45}$, ${\upgamma }_{50}$ and ${\upgamma }_{55}$ are approximately 2.00, 1.37, 1.33 and 1.22, respectively. It has been verified that the structural resistance of RC tubular structure to internal blast loading has a bi-linear behavior with an initial elastic behavior followed by a plastic behavior. Also, by implementing the internal blast correction factor ${\upgamma }$, the plastic stiffness showed almost horizontal plastic behaviour.

### RCCV simple model analysis of internal blast

**Figure 18:**
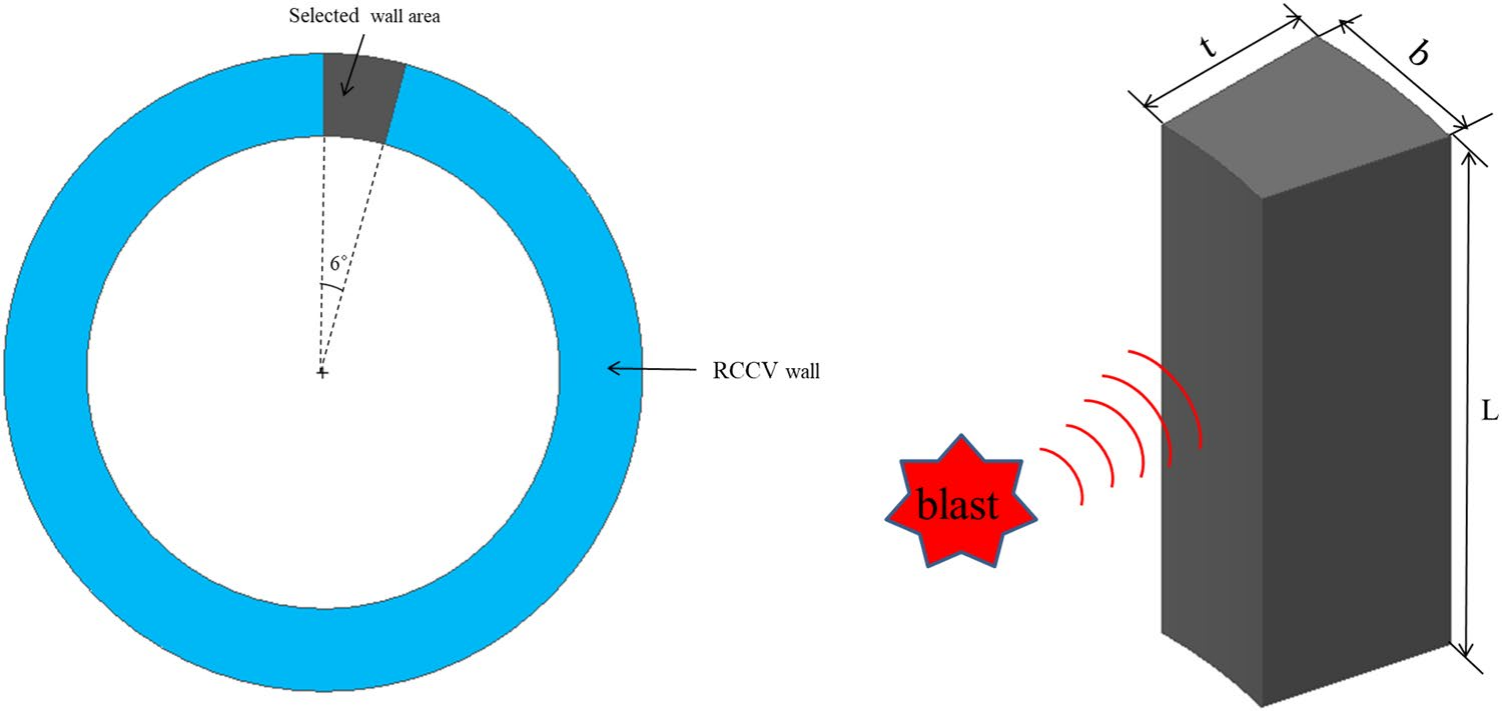
Moment of inertia calculation model.

The elastic and plastic stiffness coefficients (*K*_*el*_ and *K*_*pl*_) obtained from the test data can be used to predict the blast charge weight needed to fail a real-scale RCCV. Generally, the moment of inertia and the modulus of elasticity of contribute to the structural stiffness of a member. In order to obtain the elastic modulus of a real scale RCCV wall, the value of the moment of inertia of the model used in the experiment was obtained using Eq. (4) and dimensions shown in Fig. 18.

4 $$ I = \frac{{bt^{3} }}{12} $$

where *b* is the width of the cross-section and *t* is the thickness of the cross-section.

To correlate the member dimensions and calculate the moment of inertia for the RC specimen to a real scale RCCV wall, the dimensions were selected based on the assumptions shown in Fig. 18. As shown in the figure, a cross-sectional angle of 6° is selected to obtain the width *b*. In this study, RCCV of Kori 1 and 2 Nuclear Power Plant was selected as the target structure for the parametric analysis. The internal diameter, wall thickness, and height of the real scale RCCV and test specimen were 45,720, 1219 and 76,667 mm and 2700, 350, and 3600 mm, respectively.

At each 6 degrees of the specimen, the internal and external radius of the specimen is 1350 mm and 1700 mm, respectively, in which the value of *b* was assumed to be the value measured at the center of the wall thickness. Therefore, according to the Eq. (4), *b*_*specimen*_ is 159.698 mm and *b*_*RCCV*_ is 2457.720 mm, which gave the moment of inertia of *I*_*specimen*_ and *I*_*RCCV*_ as $5.706 \times 10^{8} {\text{ mm}}^{4}$ and $3.711 \times 10^{12} {\text{mm}}^{4}$, respectively. The elastic and plastic stiffness and deflections from the test are tabulated in Table 3. A stiffness of a wall with a free-free boundary condition is given as

5 $$ K_{el} = \frac{EI}{{L^{3} }}. $$

**Table 3:** Elastic and plastic stiffness and deflection from the test.

Type	RC35	RC45	RC50	RC55
$K_{el} (N/mm$*)*	0.65	0.65	0.65	0.65
$K_{pl}$ $(N/mm$*)*	–	0.05	0.05	0.05
$U_{el}$*(mm)*	6.5	6.5	6.5	6.5
$U_{pl}$*(mm)*	–	14.6	15.4	16.4

Since, the continuum mechanics equations can be only applied to an elastic member, the elastic behavior was assumed for the RCCV calculations. Since both the test specimen and the real scale RCCV used exactly the same materials, reinforcing bar ratio, and cross-sectional design, *E*_*specimen*_ and *E*_*RCCV*_ have to be same at elastic state assuming that there is no size effect. For the test specimen and the real scale RCCV, the stiffness is expressed as $K_{specimen}$ = $\frac{{EI_{specimen} }}{{L_{specimen}^{3} }}$, and $K_{RCCV} = \frac{{EI_{RCCV} }}{{L_{RCCV}^{3} }}$, repectively.

The curvature to applied moment relation can be expressed as

6 $$ \frac{M}{EI} = \emptyset . $$

where $\emptyset$ is the curvature of structure and *M* is the applied moment to the wall by the internal blast pressure. The maximum applied load $F_{max}$ to the wall can be calculated by multiplying the maximum blast pressure $P_{max}$ by the surface area of the $2r_{internal}$ region at the center span, as shown in Eq. (3).

Since the test setup has a boundary condition of a pin-pin condition, *M* can be calculated by.

7 $$ M = \frac{{F_{max} }}{2} \cdot \frac{L}{2}. $$

It is important to note that the wall cross-section dimensional scaling of the test specimen and the real scale RCCV is consistent. Also, the materials used for the construction and the cross-sectional design are exactly same for both the test specimen and the real scale RCCV. Therefore, the curvature which induced the test specimen failure must be equivalent to the curvature that induces a real scale RCCV failure as shown in Eq. (8).

8 $$ \emptyset_{specimen}^{fail} = \emptyset_{RCCV}^{fail} $$

By substituting Eqs. (7) and (8) into Eq. (3) and the simplifying equation, the following equations can be obtained.

9 $$ P_{max}^{RCCV} = \left( {\frac{{L_{specimen} }}{{L_{RCCV} }}} \right) \cdot \left( {\frac{{I_{RCCV} }}{{I_{specimen} }}} \right) \cdot \left( {\frac{{r_{internal}^{specimen} }}{{r_{internal}^{RCCV} }}} \right)^{2} \cdot P_{max}^{specimen} $$

Using Eq. (9), the maximum internal blast pressure required to fail a real scale RCCV can be calculated. Then using the ConWEP calculated results in Fig. 8b with an internal blast factor ${\upgamma }$ calculated previously as 2.0, the detonation charge weight can be predicated. For the RCCV of Kori 1 and 2, the required maximum internal blast pressure comes out to be 1.202 × 10^9^ MPa, which can be produced by ANFO charge weight of 766.88 kg. All of the calculated results of the test specimen and real scale RCCV are tabulated in Table 4.

**Table 4:** Calculated results of the test specimen and real scale RCCV.

Type	Test specimen	Real scale RCCV
*I* $\left[ {{\text{mm}}^{4} } \right]$	5.706$\times 10^{8}$	$3.711 \times 10^{11}$
$K_{el} \left[ {{\text{N}}/{\text{mm}}} \right]$]	0.65	91.879
*E* [N/mm^2^]	111.614	111.614
$\emptyset$	0.055667	0.055667
$M$[N∙mm]	3.54526 × 10^9^	2.30572 × 10^12^
$P_{max} \left[ {MPa} \right]$	3.93918 × 10^6^	1.20293 × 10^9^
*ANFO charge weight* [kg]	15.876	766.88

## Conclusions

In this study, the internal blast resistance capacity of RCCV was evaluated by fabricating a scaled-down model of a RCCV and conducting an experiment. The effect of the charge weight depend blast pressure on damage to the specimen was evaluated by varying the explosive charge weight from 15.88 kg to 24.95 kg. The following conclusions can be drawn from the study.

1. A RC tubular structure is fabricated by scaling down a RCCV structure to apply internal blast loading scenario. Using the scaled down specimen, the internal ANFO explosive charge weight from 15.88, 20.41, 22.68, 24.95 kg was applied to the test. The blast test data of pressure, deflection, strain, and crack pattern were obtained. In addition, a system for precise data acquisition was proposed
2. Specimens RC35, RC45, RC50, and RC55 according to the amount of explosion were designed and tested. Maximum deflection and residual deflection data were obtained according to the blast experiment of RC35, RC45, RC50, and RC55 specimen is 6.57, 14.67, 15.27, and 16.25 mm, respectively. The test data were used to calculate elastic and plastic structural of stiffness of the specimen center internal blast load, which gave the result of 0.65, and 0.05 N/mm, respectively. Since the test specimen and the real scale RCCV used exactly the same material for construction, reinforcing bar ratio and cross-sectional design, *E*_*specimen*_ and *E*_*RCCV*_ have to be same.
3. The pressuremeter data suggest that there were multiple peaks in behavior of the RC structure from an internal blast. Therefore, a more in-depth evaluation of the time dependent pressure behavior from internal blast loading in RCCV structures is needed in the future.
4. Based on simple and the test data of the deflection-force relation, the charge weight required to fail a real scale RCCV OF Kori 1 and 2 nuclear power plant in Korea is predicted as 766.88 kg.

## Data Availability

The datasets used and/or analyzed during the current study are available from the corresponding author on reasonable request.
